# Recruitment of pial collaterals and carotid occlusive disease in large-vessel occlusion ischemic stroke

**DOI:** 10.3389/fneur.2024.1423967

**Published:** 2024-10-28

**Authors:** Niklas Helwig, Marlies Wagner, Alexander Seiler

**Affiliations:** ^1^Department of Neurology, University Hospital Frankfurt (Goethe University), Frankfurt, Germany; ^2^Institute of Neuroradiology, University Hospital Frankfurt (Goethe University), Frankfurt, Germany; ^3^Brain Imaging Center, Goethe University Frankfurt, Frankfurt, Germany; ^4^Department of Neurology and Neurovascular Center, University Hospital Schleswig-Holstein, Campus Kiel, Kiel, Germany

**Keywords:** ischemic stroke, collaterals, MRI, perfusion-weighted imaging, carotid stenosis, functional outcome

## Abstract

**Background and purpose:**

Despite the fundamental role of pial collateral vessels in limiting the progression of ischemic tissue injury in acute stroke with large vessel occlusion (LVO), in addition to the fact that collateral vessel abundance varies naturally from person to person for genetic reasons, there is limited knowledge regarding potential factors contributing to inherent interindividual variation in pial collateral supply. As it has been repeatedly hypothesized that chronic carotid occlusive disease may favor pial collateralization, we aimed to investigate the association between quantitatively assessed leptomeningeal collateral supply and pre-existing carotid stenosis in patients with acute stroke due to LVO.

**Materials and methods:**

Patients with proximal middle cerebral artery (MCA) occlusion with or without additional internal carotid artery (ICA) occlusion were included. The degree of collateral supply was quantitatively assessed based on signal variance in T2*-weighted time series in perfusion-weighted magnetic resonance imaging (PWI). Patients were stratified into two groups according to quantitative collateral status (poor and fair to good collateral supply). The prevalence of high-grade ICA stenosis (≥70%) was evaluated in both groups.

**Results:**

A total of 98 patients (mean age 68.8 ± 16.1 years, *n* = 52 (53.1%) of whom were female individuals) with MCA and/or ICA occlusion were included in the final analysis. Out of these patients, 42 had poor collateral supply, while 56 exhibited fair to good collateral supply. Additionally, 18 patients showed ipsilateral high-grade ICA stenosis. After classifying the entire cohort based on their collateral status (poor vs. fair to good collateral supply), there was no significant difference in the proportion of the patients with ipsilateral high-grade ICA stenosis between the two groups. Specifically, 6 (14.3%) patients had poor collateral supply, and 12 (21.1%) patients had fair to good collateral supply. The odds ratio (OR) was 1.58, with a 95% confidence interval (CI) of 0.490–5.685 and the *p-*value of 0.440. In the entire patient cohort, signal variance-based collateral supply was significantly correlated with initial stroke severity (*r* = −0.360, *p* < 0.001), baseline ischemic core volume (*r* = −0.362, *p* < 0.001), and functional outcomes (score on the modified Rankin Scale) at discharge (*r* = −0.367, *p* < 0.01).

**Conclusion:**

In this study, we performed a quantitative and observer-independent MRI-based collateral assessment in patients with LVO. We found no significant difference in the prevalence of pre-existing high-grade ICA stenosis between patients with fair to good collateral supply and those with poor collateral supply. The potential influence of demographic and clinical variables on pial collateral supply in patients with acute stroke warrants further exploration in future studies. MRI-based collateral supply is significantly related to initial stroke severity, ischemic core volume, and early functional outcomes.

## 1 Introduction

In acute ischemic stroke (AIS) due to large vessel occlusion (LVO) of the anterior circulation, pial collaterals are key determinants of the progression rate of ischemic tissue damage and predictors of successful reperfusion, early neurological improvement, and clinical outcomes after endovascular thrombectomy (EVT) (1, 2). Despite the pivotal role of collateral vessels in stroke outcomes, there is a considerable gap in knowledge regarding the demographic and clinical factors that may favor the long-term anatomical development and recruitment of collateral vasculature in cases of acute cerebral ischemia. Previous research has yielded slightly inconsistent and conflicting results regarding the association between pial collateral supply and cardiovascular risk factors or comorbidities, and the contribution of these variables is poorly understood (2–5).

Similar to peripheral or coronary artery disease (6), chronic tissue hypoperfusion and resulting hypoxia due to atherosclerotic occlusive disease of brain-supplying arteries have been hypothesized as potential stimuli for leptomeningeal collateralization (1). Recruitment of pial collaterals is commonly considered a secondary compensatory mechanism in cerebral artery steno-occlusive disease, which is activated when the primary collateral pathways of the circle of Willis (CoW) fail to maintain sufficient blood flow to brain areas affected by chronic hypoperfusion (1, 7). High-grade internal carotid artery (ICA) occlusive disease leads to blood flow reduction and hemodynamic impairment in the ipsilateral middle cerebral artery (MCA) (8) and may be associated with the activation of secondary collaterals (7). Therefore, from a theoretical point of view, it is conceivable that pre-formed cortical anastomoses due to ICA occlusive disease may be preferably recruited in cases of acute ischemic stroke (1, 9). However, a clear association between the presence of high-grade internal carotid artery (ICA) stenosis and the degree of collateral supply in patients with acute stroke could not be demonstrated in previous studies (10). Consequently, in contrast to intracranial steno-occlusive disease (11), extracranial ICA occlusive disease may have a minor impact on the recruitment of intracranial pial collateral vessels.

Currently, collateral imaging in the clinical acute setting largely relies on the use of approximative and observer-dependent grading systems, making the results susceptible to imprecision and less sensitive to interindividual variation in collateral flow (12). In contrast, recently developed collateral imaging based on signal variance in perfusion-weighted magnetic resonance imaging (MRI) enables the generation of a continuous variable for a (semi-)quantitative and rater-independent assessment of collateral supply (13, 14). In this study, we aimed to investigate the association between pial collateralization and the presence of carotid occlusive disease in patients with acute large vessel occlusion (LVO) by utilizing signal variance in perfusion-weighted imaging (PWI) as the basis for collateral assessment.

## 2 Materials and methods

### 2.1 Patients

**Figure 1:**
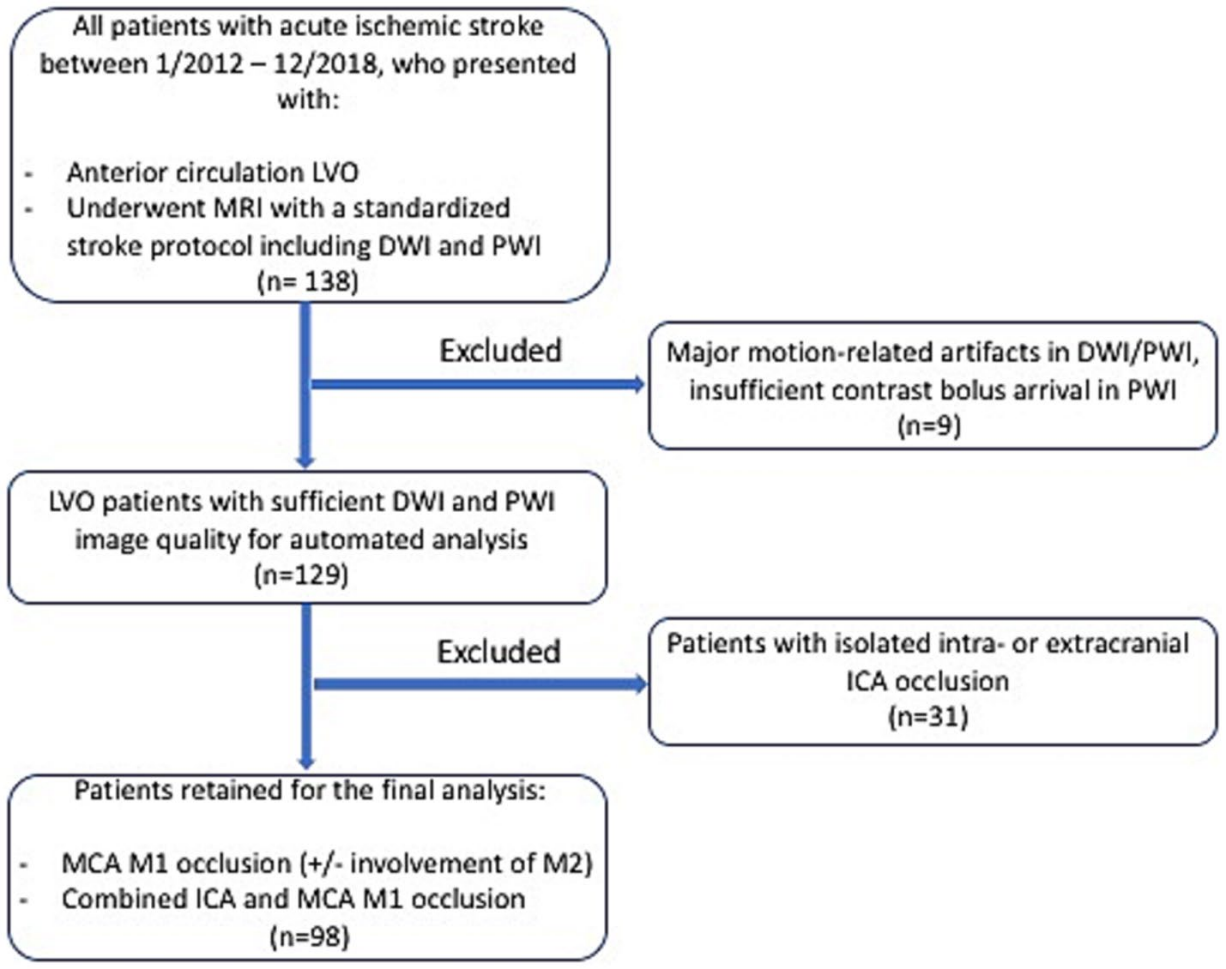
Flowchart illustrating the stepwise inclusion of the patients for the retrospective study. LVO, large-vessel occlusion; MRI, magnetic resonance imaging; DWI, diffusion-weighted imaging; PWI, perfusion-weighted imaging; ICA, internal carotid artery; MCA, middle cerebral artery.

Patients with LVO of the anterior circulation who underwent a standardized stroke MR imaging protocol—including diffusion-weighted imaging (DWI) and PWI—with sufficient image quality (complete contrast bolus arrival in PWI; no major motion-related artifacts in DWI and PWI) for automatic image analysis, including ischemic core segmentation and fully automated PWI-based collateral assessment, were retrospectively selected from the clinical imaging database of the Institute of Neuroradiology, University Hospital Frankfurt (Goethe University), Frankfurt, Germany. From 2012 to 2018, 138 patients with LVO were identified. Nine patients were excluded due to motion-related artifacts in DWI and/or PWI or insufficient bolus arrival in PWI. Of the remaining 129 patients, 31 were excluded due to isolated extracranial or intracranial ICA occlusion. This approach was chosen because, in cases with isolated ICA occlusion, the principal route of anterograde blood flow to the MCA territory remains patent, which confounds the assessment of retrograde flow to the affected vascular territory via leptomeningeal collaterals (10). Consequently, 98 patients with isolated MCA M1 occlusion (with or without extension to the M2 segments) or combined ICA and MCA M1 occlusion were included in the final analysis. The details of patient inclusion and stepwise exclusion are outlined in Figure 1. The study was approved by the local institutional review board at Goethe University Frankfurt, Faculty of Medicine (approval number: 400/18). Informed consent from the individual patients was waived because of the retrospective nature of the study.

### 2.2 MR imaging protocol

DWI data were acquired using a single-shot spin-echo echo-planar imaging (EPI) sequence with the following parameters: echo time TE = 88 ms, repetition time TR = 4,900 ms, flip angle 90°, field-of-view 220 × 220 mm^2^, matrix size 130 × 130, 25 axial slices, slice thickness 5 mm, inter-slice gap 0.5 mm, and bandwidth BW = 1,425 Hz/pixel. Diffusion-sensitizing gradients were applied sequentially with b = 0, b = 500 s/mm^2^, and b = 1,000 s/mm^2^. Apparent diffusion coefficient (ADC) maps were calculated using commercially available scanner software. Perfusion-weighted images were acquired using a T2*-weighted dynamic susceptibility contrast (DSC) gradient-echo EPI sequence. Imaging parameters were as follows: TE = 30 ms, TR = 1,500 ms, flip angle FA = 90°, field-of-view 230 × 230 mm^2^, matrix size 128 × 128, 19 axial slices, slice thickness 4 mm, 1.2 mm inter-slice gap, BW = 1,447 Hz/pixel, and acquisition time 1:41 min. The intravenous contrast agent gadobutrol (0.1 mmol/kg; Gadovist^®^ Bayer) was automatically administered by a power injector at a flow rate of 5 mL/s, followed by a bolus (20 mL) of 0.9% saline.

### 2.3 Image postprocessing and analysis for ischemic core volumetry and quantitative assessment of collateral supply

Further image postprocessing and analysis were performed automatically using in-house-built shell and MATLAB scripts, implementing tools provided in the FMRIB’s Software Library toolbox (FSL, version 5.0.7^1^).

The ischemic core at admission was defined using an established and validated upper threshold of 620 × 10^−6^ mm^2^/s on apparent diffusion coefficient (ADC) maps (15). Each automatically segmented ADC lesion was thoroughly inspected for accuracy and manually corrected if necessary. During this procedure, the corresponding diffusion-weighted image (b = 1,000 s/mm^2^) was taken into consideration. The segmented infarct core was used for volumetric assessment.

**Figure 2:**
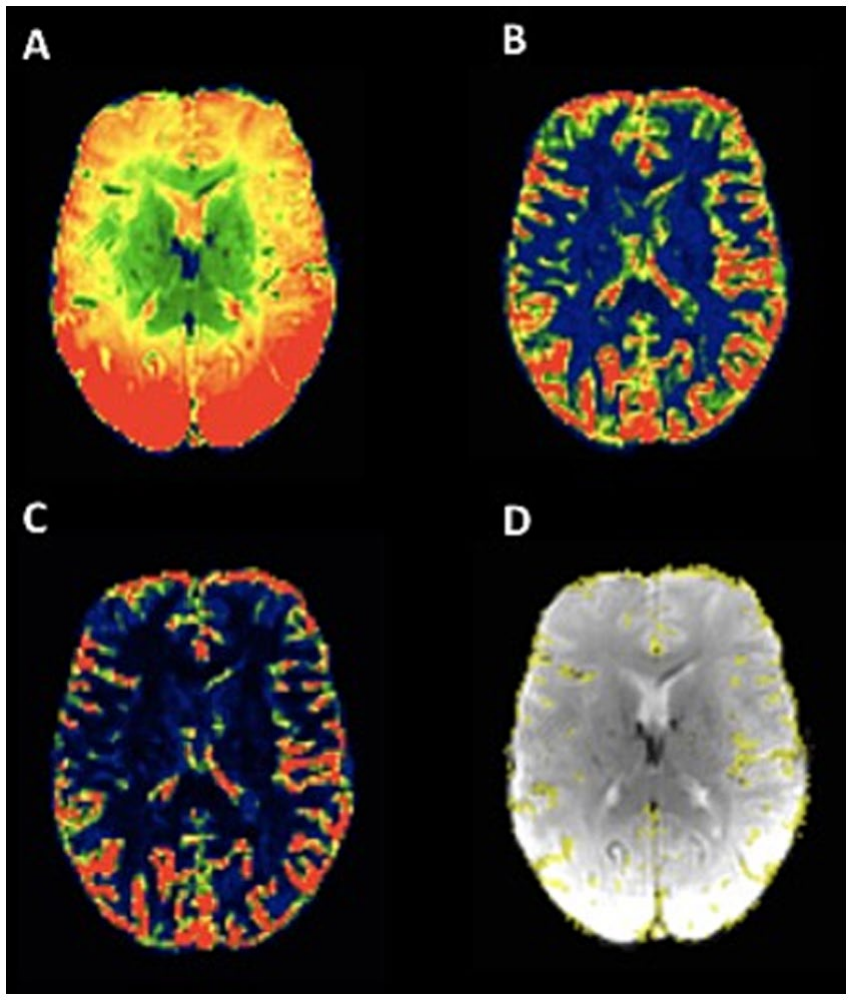
Illustration of pial collateral vessel maps based on signal variance over time in the T2*-weighted PWI time series. A: mean signal over time. B: signal standard deviation over time. C: signal variance over time, obtained from the voxel-wise division of the standard deviation by the mean. D: map of the pial collateral vasculature computed based on signal variance, with a standardized correction procedure, overlaid on the first volume of the PWI time series (acquired as part of the clinical standard stroke MR imaging protocol) after the brain extraction. The images were obtained from a patient with right-sided MCA occlusion, and the collateral vessel map shows slightly reduced collateral supply on the side of LVO.

For an automated quantitative and observer-independent assessment of collateral supply, T2*-weighted PWI time series were processed as described in detail recently (13, 14). After the motion correction of the T2*-weighted time series and the calculation of maps representing voxels with a high coefficient of variance (CoV) in the signal-time curves, as well as after applying established thresholding, maps of the pial collateral vasculature were computed using a correction procedure (Figure 1). In brief, images representing the mean signal intensity (Figure 2A) and the standard deviation over time (Figure 2B) in the T2*-weighted PWI time series were created to obtain the CoV map (calculated as the standard deviation divided by the mean, Figure 2C), which was then further processed to create a map of the pial collateral vasculature (Figure 2D). Finally, based on the latter map, a collateral vessel index (CVI_PWI_) was calculated by dividing the volumetric abundance of the collateral vessels along the lateral and cranial convexity of the affected side by that of the unaffected side. The detailed procedure for the automated quantitative collateral assessment using the PWI time series has been described elsewhere (14).

### 2.4 Demographic and clinical characteristics and image evaluation/analysis

Demographic baseline characteristics and data on vascular risk factors and stroke etiology of all patients were collected. For the assessment of clinical stroke severity and early functional outcomes, scores on the National Institutes of Health Stroke Scale (NIHSS) were recorded at three time points (upon admission, at 24 h, and at discharge) and the individual score on the modified Rankin Scale (mRS) was obtained at discharge. An assessment of ICA stenosis was performed based on digital subtraction angiography (DSA) or CT angiography (CTA), using the NASCET criteria. With regard to the degree of stenosis, strong correlations have been demonstrated between the two imaging methodologies (16). Time-of-flight (TOF) MR angiograms were systematically evaluated for the completeness of the Circle of Willis (CoW), focusing on the presence of the anterior communicating artery, the A1 segments of the anterior cerebral artery (ACA), and the patency of the posterior communicating arteries and the P1 segments of the posterior cerebral artery (PCA). Image evaluation was performed by an experienced neuroradiological reader who was blinded to all other clinical and imaging data. The patient cohort was subdivided into two groups (fair to good vs. poor collateral supply) after stratification according to a previously established CVI_PWI_ threshold of 0.96 (14).

### 2.5 Statistical analysis

Categorical variables were described using counts and percentages, while continuous and ordinal variables were described as mean ± SD or median and interquartile range. Proportions were calculated using χ^2^ statistics, whereas continuous and ordinal variables were compared using the Mann–Whitney U test or *t*-test, depending on the distribution of the data. In addition to the presence of ipsilateral or contralateral high-grade ICA stenosis, associations between collateral supply and the presence of any ICA stenosis ≥50% were investigated in subanalyses. Due to the low overall number of cases with ICA stenosis, Fisher’s exact test was used for group comparisons of this variable. The relationship between pial collateral supply (CVI_PWI_) and ischemic core volume, as well as stroke severity (NIHSS on admission, at 24 h, and at discharge) and early functional outcomes (mRS at discharge), was investigated using a covariate-adjusted correlational analysis. A binary logistic regression analysis was performed with age, sex, and the completeness of the Circle of Willis (CoW) as covariates to calculate the adjusted odds ratio (OR) and 95% confidence interval (CIs) for ipsilateral and contralateral high-grade ICA stenosis in relation to favorable collateral supply. Statistical analyses were performed using JASP 0.18.3 (The University of Amsterdam^2^).

## 3 Results

A total of 98 patients (mean age 68.8 ± 16.1 years, *n* = 52 (53.1%) of whom were female individuals) with MCA occlusion with or without additional ICA occlusion were included in the final analysis. Stratification according to collateral status based on an established CVI_PWI_ threshold yielded a group of 56 patients with fair to good collateral supply and a group of 42 patients with poor collateral supply.

The patients with fair to good collateral supply did not differ from the patients with poor collateral supply in terms of age, sex, stroke etiology, cardiovascular risk factors, comorbidities, time from symptom onset to imaging, occlusion laterality, and site of LVO (Table 1). The proportion of the patients receiving intravenous thrombolysis and achieving successful reperfusion (defined as the thrombolysis in cerebral infarction (TICI) score ≥ 2b) was similar in both groups (Table 1). The patients with fair to good collateral supply had significantly lower baseline NIHSS scores upon admission and lower baseline ischemic core volume compared to the patients with poor collateral supply (*p* ≤ 0.001). The proportion of the patients with the complete CoW was significantly higher in the group with fair to good collateral supply (89.3% vs. 73.8%, *p* = 0.045). Furthermore, the patients with fair to good collateral supply exhibited significantly better early functional outcomes (NIHSS and mRS at discharge) (*p* = 0.002, Table 1). Ipsilateral high-grade ICA stenosis was present in *n* = 6 (14.3%) cases in the poor collateral group and in *n* = 12 (21.1%) in the group with fair to good collaterals (OR = 1.58, 95% CI 0.490–5.685, *p* = 0.440). Instead of atherothrombotic etiology, cardioembolism was identified as the underlying etiology in 1 patient with high-grade ICA stenosis ipsilateral to ischemic stroke. Contralateral high-grade ICA stenosis was present in 2 (4.8%) patients in the poor collateral group and in 4 (7.1%) patients in the group with fair to good collaterals (OR = 1.53, 95% CI 0.208–17.726, *p* = 0.698). The extension of the criterion for ipsilateral and contralateral ICA stenosis to a degree of ≥50% did not result in any significant group differences regarding the presence of ICA stenosis (*p* = 0.721 and *p* = 0.649).

**Table 1:** Results of the univariate analysis after the stratification of the patient cohort according to collateral status.

	Poor collaterals (CVI_PWI_ < 0.96)	Fair to good collaterals (CVI_PWI_ ≥ 0.96)	*p*-value
*n*	42	56	
Age [mean ± SD]	69.3 ± 15.9	67.9 ± 16.4	0.550
Female sex [*n* (%)]	22 (52.4)	30 (53.6)	0.907
**Cardiovascular risk factors and comorbidities [*n* (%)]**			
Arterial hypertension	26 (61.9)	38 (67.9)	0.540
Diabetes	6 (14.3)	10 (17.9)	0.636
Smoking	13 (31.0)	11 (19.6)	0.198
Hyperlipidemia	8 (19.0)	7 (12.5)	0.373
Atrial fibrillation	14 (33.3)	14 (25.0)	0.366
Coronary artery disease	3 (7.1)	7 (12.5)	0.386
Cardiac insufficiency	3 (7.1)	9 (16.1)	0.182
Onset-to-imaging time [minutes] (mean ± SD)	432 ± 329	476 ± 338	0.541
**Stroke etiology [*n* (%)]**			0.624
Cardioembolic	16 (38.1)	18 (32.8)	
Large-artery disease	14 (33.3)	21 (38.2)	
Other	3 (7.1)	4 (7.2)	
Unknown	9 (21.5)	12 (21.8)	
Occlusion laterality, right [*n* (%)]	18 (42.9)	34 (60.7)	0.080
**Occlusion site [*n* (%)]**			0.342
MCA M1	34 (81.0)	48 (85.7)	
ICA + MCA M1	8 (19.0)	8 (14.3)	
Complete CoW [*n* (%)]	31 (73.8)	50 (89.3)	**0.045**
Ipsilateral high-grade ICA stenosis [*n* (%)]	6 (14.3)	12 (21.1)	0.440
Contralateral high-grade ICA stenosis [*n* (%)]	2 (4.8)	4 (7.1)	0.698
NIHSS admission [median (IQR)]	14 (17.75–9)	10 (14–4)	**<0.001**
Ischemic core volume admission [cm^3^] (mean ± SD)	39.0 ± 40.1	19.6 ± 16.2	**0.001**
i.v. thrombolysis [*n* (%)]	18 (42.9)	22 (40.0)	0.837
EVT [*n* (%)]	29 (69.0)	35 (62.5)	0.398
Recanalization TICI 2b+[*n* (%)]	20 (47.6)	23 (41.1)	0.812
NIHSS at 24 h [median (IQR)]	14 (7.5–19.5)	6.5 (4–11.5)	**0.009**
NIHSS discharge [median (IQR)]	12.5 (4.75–42)	4 (1–8)	**0.002**
mRS discharge [median (IQR)]	4 (3–5)	2.5 (2–4)	**0.002**

CVI_PWI_, Collateral vessel index in perfusion-weighted imaging; SD, standard deviation; mRS, modified Rankin Scale; MCA, middle cerebral artery; ICA, internal carotid artery; CoW, circle of Willis; NIHSS, National Institutes of Health Stroke Scale (NIHSS); cm^3^, cubic centimeters; IQR, interquartile range; EVT, endovascular thrombectomy; TICI, Thrombolysis in Cerebral Infarction.

After adjusting for age, sex, and the completeness of the CoW as covariates, the OR for ipsilateral ICA stenosis of ≥70% for fair to good collateral supply was 1.61 (95% CI 0.469–5.523, *p* = 0.449) in the binary logistic regression analysis. For contralateral ICA stenosis of ≥70%, the OR for fair to good collateral supply was 1.33 (95% CI 0.178–9.951, *p* = 0.781). There was a trend to statistical significance for the association between the complete CoW and fair to good collateral supply in the binary logistic regression analysis: OR 2.802 (95% CI 0.901–8.711, *p* = 0.075) (Table 2). The comparison of the CVI_PWI_ values (as a quantitative measure of collateral supply) of the patients with ipsilateral (*n* = 18) and contralateral (*n* = 6) high-grade ICA stenosis with the CVI_PWI_ values of the respective remaining patient cohort did not reveal any significant group differences (*p* = 0.767 and *p* = 0.762).

**Table 2:** Results of the binary logistic regression analysis for the fair to good collateral supply (defined as a CVI_PWI_ value ≥ 0.96).

Parameter	Odds ratio (95% CI)	*p*-value
Age	0.994 (0.968–1.020)	0.630
Sex (female)	1.267 (0.540–2.972)	0.587
Complete CoW	2.802 (0.901–8.711)	0.075
Ipsilateral high-grade ICA stenosis	1.610 (0.469–5.523)	0.449
Contralateral high-grade ICA stenosis	1.331 (0.178–9.951)	0.781

CI, confidence interval; CoW, circle of Willis; ICA, internal carotid artery.

**Figure 3:**
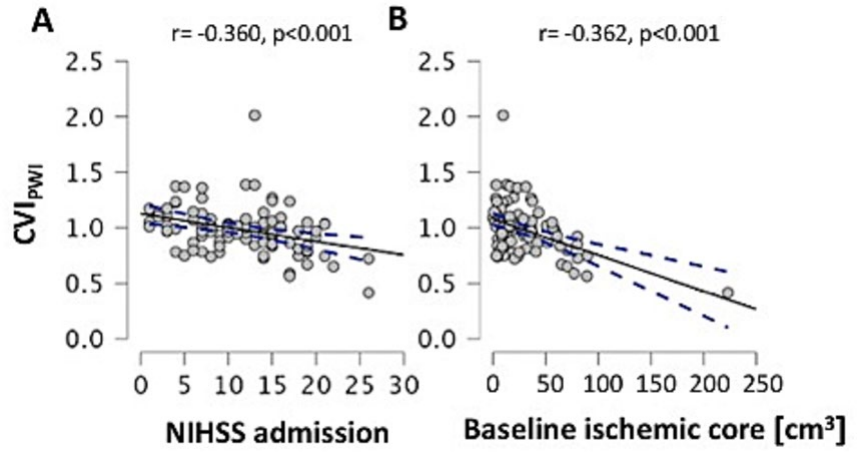
Scatterplots illustrating the relationship of leptomeningeal collateral supply with NIHSS on admission (A) and baseline ischemic core volume (B) after controlling for age. CVI_PWI_: Collateral vessel index in perfusion-weighted imaging; NIHSS: National of Institute of Health Stroke Scale; cm^3^: cubic centimeters. A line of best fit and the 95% confidence interval are shown in each graph.

Across the entire patient cohort, the CVI_PWI_ was significantly correlated with the NIHSS on admission (*r* = −0.360, *p* < 0.001, Figure 3A) and baseline ischemic core volume (*r* = −0.362, *p* < 0.001, Figure 3B) when adjusting for age. After adjusting for the covariates of age, baseline ischemic core volume, and successful recanalization after EVT (TICI ≥ 2b), the CVI_PWI_ was significantly correlated with the NIHSS at 24 h (*r* = −0.419, *p* = 0.015), the NIHSS at discharge (*r* = −0.335, *p* = 0.028), and the mRS at discharge (*r* = −0.367, *p* = 0.004).

## 4 Discussion

In this study, we used an objective, rater-independent, and (semi-)quantitative method with a continuous variable for assessing collateral flow, based on signal variance in the T2*-weighted time series of MR-PWI. This method is independent of inter-observer variation and grading scales (13, 14) and was used to evaluate the association between pre-existing high-grade ICA stenosis and pial collateral supply in patients with anterior circulation LVO. Our results did not reveal a significant association between pial collateral supply and pre-existing stenosis of the ipsilateral or contralateral ICA (Tables 1, 2). However, we found correlations of better pial collateral supply (higher CVI_PWI_) with lower ischemic core volume, less severe stroke, and better functional outcomes at discharge, after adjusting for the covariates age and successful reperfusion (Figure 3).

It has been repeatedly hypothesized that pre-existing atherosclerotic steno-occlusive disease may favor the recruitment of collateral vessels (1, 9). This could have potential clinical implications with regard to limiting ischemic core expansion, increasing the possibility of a favorable outcome in acute ischemic stroke, and making therapeutic approaches to further enhance collateral flow more promising. Our results are in contrast with a previous study that described increasing collateralization with an increasing degree of stenosis in patients with symptomatic intracranial atherosclerotic stenosis, based on intraarterial angiography (11). However, this study included a heterogeneous population with various sites of stenosis and without acute LVO (11). For proximal ICA stenosis, Dankbaar et al. did not demonstrate a significant association with ipsilateral collateral supply in LVO when using CT perfusion-derived vessel images and a time-resolved method for collateral assessment. In contrast, a more recent study observed an independent association between pre-existing ICA stenosis and leptomeningeal collaterals in patients with MCA occlusion (17). This study used standard single-phase CTA for collateral grading, which is not time-resolved and therefore represents a less reliable method for assessing the actual magnitude of collateral flow, especially regarding late-filling smaller collateral vessels (18). In contrast to the (semi-)quantitative approach employed in the present work, both studies used more approximate and operator-dependent rating scales to assess the magnitude of collateral supply (10, 17). Together with the technical differences of the employed imaging modalities, this may explain the heterogeneous results. While a positive effect on leptomeningeal collateralization could be hypothesized for ipsilateral ICA stenosis, one could expect a negative effect from high-grade contralateral ICA stenosis, leading to a reduction in intracranial blood flow and therefore potentially limiting the possibility of collateral filling from the hemisphere contralateral to LVO. This is especially relevant considering that previous studies have suggested that the magnitude of cerebral blood flow on the contralateral unaffected side represents a surrogate for collateral flow and may modify the functional outcome in patients with LVO (19). Overall, the results of our study do not suggest a significant impact of pre-existing ipsilateral or contralateral ICA on the recruitment of leptomeningeal collaterals in patients with acute stroke due to LVO. It is well-documented that collateral flow is closely related to the hemodynamic status of the brain parenchyma. Previous studies have demonstrated impaired cerebrovascular reactivity associated with leptomeningeal collateral activation in patients with symptomatic ICA occlusion (20). Given this finding, one might suspect that patients with high-grade ICA stenosis could recruit some pial collateral vessels over time, which might not necessarily be sufficiently protective in the event of acute LVO. However, a significant impairment of cerebrovascular reactivity as a surrogate of hemodynamic compromise could not be demonstrated in patients with high-grade ICA stenosis (21). Consequently, due to a less pronounced hemodynamic compromise compared to ICA occlusion, collateral recruitment in patients with ICA stenosis of ≥70% might occur to a lesser extent, preventing the detection of significantly augmented collateral supply via leptomeningeal collaterals in patients with high-grade ICA stenosis in the event of LVO. Combining collateral mapping with imaging techniques that measure cerebrovascular reactivity/cerebral autoregulation as surrogate markers of hemodynamic impairment might allow for a more detailed exploration of this question.

In addition, regarding cardiovascular risk factors as the underlying etiology of atherosclerotic ICA steno-occlusive disease and the degree of collateral supply in acute stroke, findings in the literature have been inconsistent. While one study has reported a significant association between metabolic syndrome with its associated risk factors and increased collateral supply (3), other studies have described a detrimental effect of those risk factors on leptomeningeal collaterals (22–24). In accordance with the latter finding, a growing body of literature suggests an association between risk factor-related cerebral small vessel disease and poorer collateral supply in patients with AIS (25, 26). It remains unclear whether the potential pre-formation of pial collaterals influenced by vascular risk factors, cerebral artery atherosclerosis, and preceding ischemic events is relevant for improving and prolonging the brain’s ischemic tolerance in the case of acute LVO. In fact, previous studies have suggested that leptomeningeal collaterals in acute MCA occlusion may open rapidly and be recruited immediately once a significant drop of perfusion pressure occurs in the distal MCA, leading to retrograde perfusion via cortical anastomoses (27). Although the present study does not demonstrate a contribution of cerebral atherosclerotic macroangiopathy to pial collateral supply, (semi-)quantitative mapping of collateralization using the employed methodology is related to early functional outcomes after proximal MCA occlusion (Table 1) and may be a promising approach for investigating factors associated with pial collateral supply in future studies (14).

Our study only included patients with MCA M1 occlusion (with or without additional ICA occlusion), thereby excluding cases with vessel occlusion affecting exclusively the extracranial or intracranial ICA. This exclusion consequently removed the possibility of anterograde flow to the MCA territory via the CoW and the primary route of blood supply. The latter would have complicated or confounded the assessment of pial collateral supply considerably. MCA occlusion has been linked to worse collateralization of the MCA vascular territory compared to ICA occlusion (28), which can be explained by the fact that the primary route of perfusion is not patent, limiting the possibility of pial collateralization from the ACA and the PCA territories. The patients with fair to good pial collateral supply exhibited a significantly higher proportion of the patients with the complete CoW (Table 1). Regarding the association between CoW completeness and stroke outcomes in patients with LVO, previous studies have yielded inconsistent results (29–32). However, the anatomy of the CoW may modify stroke risk and the degree of collateral supply (10, 33). Consistent with this notion, we found an association between the complete CoW and better collateral supply (Table 1), most likely reflecting pial collateral flow to the MCA territory via cortical anastomoses from the ACA and the PCA territories.

In contrast to computed tomography (CT) and MR angiography, which are the most widely applied methods for detecting LVO and collateral flow in acute stroke (12), a CVI_PWI_ relies on a time-resolved imaging method that also captures late collateral filling of smaller anastomoses and thus does not carry the risk of systematically underestimating leptomeningeal collateralization (13, 14). While the latter property of the CVI_PWI,_ along with its (semi-)quantitative and rater-independent characteristics, is certainly an important advantage of the proposed methodology, which explains the relationship between the CVI_PWI_ and baseline ischemic core volume, as well as markers of clinical stroke severity and functional outcomes (Table 1; Figure 3), its shortcomings include the rather static representation of collateral supply and the fact that it does not allow for assessing the functional properties of the individual collateral vasculature, including the magnitude of collateral blood flow and other collateral hemodynamic features. These aspects might be relevant for the overall protective effect of collateral perfusion in acute stroke. Therefore, PWI-based collateral mapping can be considered a complementary method to other imaging techniques, including transcranial Doppler ultrasound (34) and quantitative magnetic resonance angiography (qMRA) with non-invasive optimal vessel analysis (NOVA) (35, 36). Both methods allow for measuring blood flow velocities and quantifying cerebral blood flow in the arteries of the CoW, providing information about collateral blood flow, including cross-flow between cerebral hemispheres and collateral supply to adjacent vascular territories via cortical anastomoses. QMRA can be integrated into a clinical stroke imaging protocol, can be easily implemented on any MRI scanner vendor, and serves as a promising adjunct imaging method to clinical imaging paradigms for the investigation of acute and chronic cerebral hypoperfusion (35, 36).

### 4.1 Limitations

The primary limitation of this study is the relatively small sample size. The proportion of patients with ipsilateral ICA stenosis in this study is comparable to similar cohorts in previous studies (10). However, the overall number of cases is low, which might have influenced our results. In addition, given the single-center, retrospective design with data acquired in clinical routine over a period of 6 years, the results of this study should be interpreted and generalized with caution. Even though MR imaging techniques for stroke imaging in the acute setting have not changed fundamentally in recent years, the inevitable optimization of imaging sequences may limit the generalizability of our findings. In addition, this study was not powered to conduct dedicated subgroup analyses on the association between various degrees of ICA stenosis and leptomeningeal collateral vessel abundance. Furthermore, with the data available in this study, we cannot comment on the chronicity of the stenoses detected in the current patient cohort. The chronicity may be a decisive factor in the recruitment of pial collaterals since more established carotid occlusive disease with chronic cerebral hypoperfusion may stimulate collateralization with a higher probability than recently developed and potentially more rapidly progressing ICA stenosis. Due to the lack of more detailed clinical data, such as blood pressure values at the time of admission, the available data do not allow for more comprehensive analyses regarding the influence of various demographic and clinical factors on collateral supply. As collateral failure may be triggered by acute blood pressure changes, this would be an important variable to investigate in future studies. Finally, the results obtained from PWI-based collateral imaging could not be compared to an intra-arterial angiography-based assessment of pial collateralization, which is still considered the gold standard of collateral imaging.

## 5 Conclusion and future directions

In this study, no association could be found between quantitative and observer-independent collateral supply and pre-existing high-grade ICA stenosis in the patients with LVO. The exact pathophysiological background of collateral recruitment in acute stroke warrants further exploration in future studies. PWI-based collateral mapping might be a promising method to investigate factors associated with pial collateral supply in acute ischemic stroke. Due to the somewhat conflicting results and significant current uncertainties regarding the factors that favor the development and recruitment of collaterals in acute ischemic stroke (3, 22–24), more quantitative, rater-independent methods are highly necessary to investigate the human collaterome and perform accurate phenotyping of collateral profiles for clinical stroke trials. The results of recent randomized thrombectomy trials have shown the benefit of endovascular treatment in late-window patients with any collateral flow observed on single-phase computed tomography angiography (CTA) (37, 38). In light of these findings, the methodology employed in this study could be used to assess the degree of collateral supply in the advanced time window more reliably, helping to identify patients who are likely to benefit from recanalization more accurately. Ideally, PWI-based collateral mapping could be combined with imaging methods, allowing for the assessment of microvascular perfusion properties and metabolic tissue characteristics before and after recanalization (39–41). As collateral vessels seem to guide reperfusion at the tissue level, a critical factor for the successful preservation of tissue at risk during thrombectomy (42), this represents an intriguing question to investigate in future studies. Furthermore, PWI-based collateral mapping could be used to perform risk stratification based on collateral supply in patients with chronic cerebral hypoperfusion.

## Data Availability

The original contributions presented in the study are included in the article/supplementary material, further inquiries can be directed to the corresponding author.

## Footnotes

^1^http://www.fmrib.ox.ac.uk

^2^https://jasp-stats.org
